# Human Interaction Smart Subsystem—Extending Speech-Based Human-Robot Interaction Systems with an Implementation of External Smart Sensors

**DOI:** 10.3390/s20082376

**Published:** 2020-04-22

**Authors:** Michal Podpora, Arkadiusz Gardecki, Ryszard Beniak, Bartlomiej Klin, Jose Lopez Vicario, Aleksandra Kawala-Sterniuk

**Affiliations:** 1 Faculty of Electrical Engineering, Automatic Control and Informatics—Opole University of Technology, 45-758 Opole, Poland; a.gardecki@po.edu.pl (A.G.); r.beniak@po.edu.pl (R.B.); kawala84@gmail.com (A.K.-S.); 2 Weegree Sp. z o.o. S.K., 45-018 Opole, Poland; b.klin@weegree.com; 3 Wireless Information Networking Group, Escola d’Enginyeria—Universitat Autonoma de Barcelona, 08193 Bellaterra, Spain; jose.vicario@uab.cat

**Keywords:** pepper robot, sensor networks, smart infrastructure, humanoid robots, cloud services, thermal imaging, COVID-19

## Abstract

This paper presents a more detailed concept of Human-Robot Interaction systems architecture. One of the main differences between the proposed architecture and other ones is the methodology of information acquisition regarding the robot’s interlocutor. In order to obtain as much information as possible before the actual interaction took place, a custom Internet-of-Things-based sensor subsystems connected to Smart Infrastructure was designed and implemented, in order to support the interlocutor identification and acquisition of initial interaction parameters. The Artificial Intelligence interaction framework of the developed robotic system (including humanoid Pepper with its sensors and actuators, additional local, remote and cloud computing services) is being extended with the use of custom external subsystems for additional knowledge acquisition: device-based human identification, visual identification and audio-based interlocutor localization subsystems. These subsystems were deeply introduced and evaluated in this paper, presenting the benefits of integrating them into the robotic interaction system. In this paper a more detailed analysis of one of the external subsystems—Bluetooth Human Identification Smart Subsystem—was also included. The idea, use case, and a prototype, integration of elements of Smart Infrastructure systems and the prototype implementation were performed in a small front office of the Weegree company as a decent test-bed application area.

## 1. Introduction

Novelty and innovation are some of the most important features of a successful company and therefore companies not only pursue popular trends and cutting-edge technology, but also try to convince their customers regarding their modernity and innovation in any possible way. This may be one of the reasons why many of them try to showcase or at least advertise using futuristic technology, such as inter alia machines and robots.

WeegreeOne, a humanoid employee built upon the SoftBank Robotics’ Pepper robot, is a good example of technology implemented not only in order to bring benefits to a company as an employee, but also to be seen and perceived as the top of the innovation, served to the company’s customers.

The days, when simply having a robot was impressive, are gone. The technology should be more than just presenting content. It should be truly interactive. The authors of this work evaluated methods for the purpose of measurement and influencing the Human-Robot interaction User Experience, (see Reference [1]), concluding that the robot’s interaction is perceived as more natural if the robot is able to personalize its communication content towards a particular interlocutor. It became clear, that the system should be able to get as much information as possible during and before the conversation takes place [2]. One of the key aspects in a healthy conversation is emotional state recognition. All the improvements of the robots presented in this paper are aiming for natural, real integration with human customers with the implementation of Artificial Intelligence [3,4].

The authors of this work have indicated extending the robot’s capabilities by adding additional external wireless sensors in Reference [2], however without Smart Infrastructure. Implementation of a remote PID (Passive Infrared Detector) sensor in order to get a signal “someone is coming” seemed to be enough at that stage.

In this paper an extended approach is being presented, introducing Human Identification Smart Subsystems (HISS)—a set of external sensors, which enable us to obtain much more valuable information, resulting from the communication with the Smart Infrastructure [5] or databases of the customer. The idea presented in this paper has been successfully verified and implemented and the conclusions were here in detail presented. The HISS are able to provide detailed verified and profiled information on the interlocutor, before the actual interaction takes place. Therefore, the use of preliminary identification and categorization mechanisms during and before verbal human-machine interaction can provide a sense of temporal adequacy when initiating contact with the system, greatly improving the overall User Experience of interlocutors. The features, which can be acquired by the proposed HISS subsystems during the pre-interaction stage (regarding some of the characteristics of a particular interlocutor), as well as the fact of their earlier acquisition, accelerates and improves identification of the interlocutor.

Currently, many companies need to manage extensive IT (Information Technology) systems, customer databases and transaction databases. However, these systems can be used with a larger business effect, as well as greater marketing impact, by combining them with the Smart Infrastructure sensing conception. Company databases and information systems seen and presumed as its information resources infrastructure, can and should be understood as the source data to be integrated into the dynamic dialogue trees generated in run-time by robots’ AI (Artificial Intelligence) framework. This strategy combines the sensing technologies embedded in infrastructure and the equipment it interacts with. Real-time data acquisition and analysis in conjunction with the information already gathered, results in better interpretation of the data [1], which is clearly visible in inter alia numerous Internet of Things (IoT)-based healthcare applications [6] or in robotics [7], or in autonomous systems [8]. The use of a humanoid robot gives additional benefits in building the company’s relationship with a client [4,7,9,10,11].

The main aim of this work was to extend humanoid robots, which could be considered as both social and service robots using various smart sensors. Another innovation in this work is the HISS system, its idea, design and prototype implementation, fully developed by the authors of this work. It also covers numerous modes of interactions including verbal, non-verbal and sensor-based.

## 2. Materials and Methods

This paper proposes a system, which combines the Smart Infrastructure concept with not only the AI framework but also the individual smart sensor subsystems.

**Figure 1:**
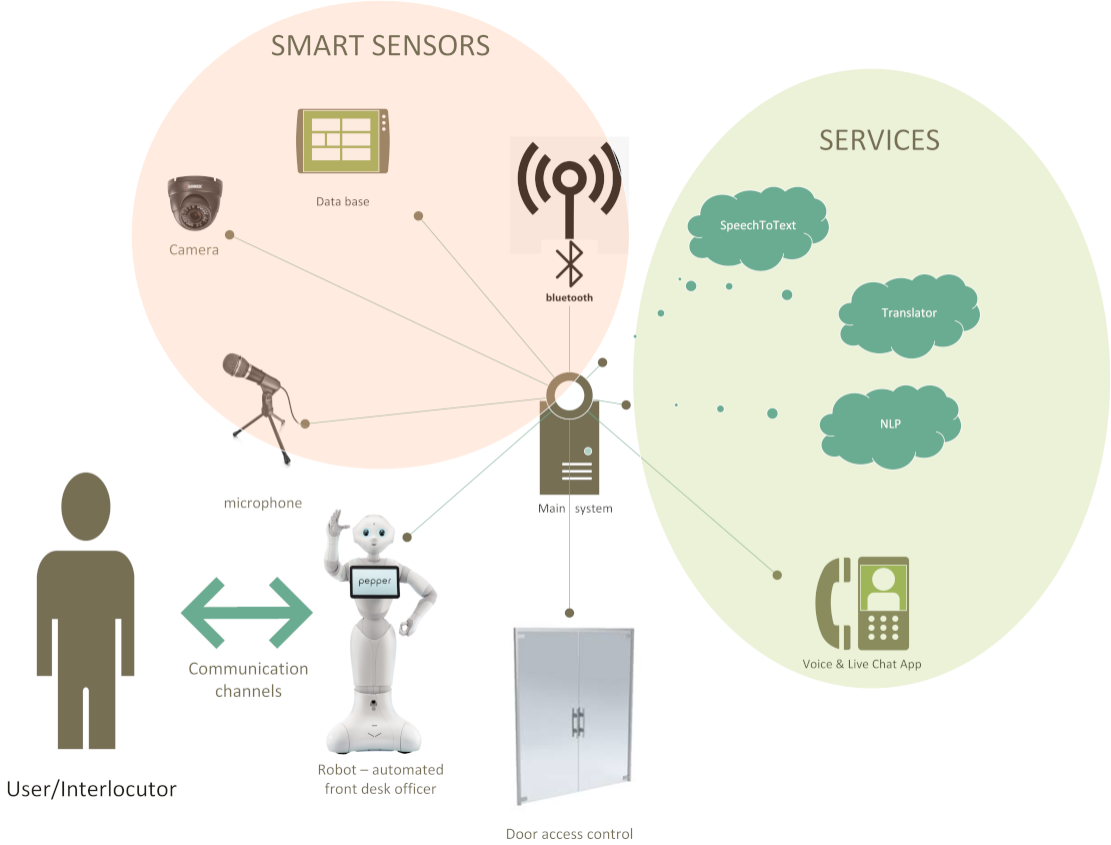
Simplified overview diagram of the proposed system’s components.

Figure 1 presents a simplified diagram of the proposed system’s components. It consists of numerous sensors, actuators, interaction subsystems, computing services (local, remote and cloud services) and databases. The part of the Figure 1 named ”smart sensors” depicts some of the information sources (which can be chosen from a set of designed sensor nodes, such as for example, Bluetooth (BT) sensor node, designed in order to interact with the Smart Infrastructure (mainly databases) of the company to convert the raw data into a useful high-level personalized information. This is in part similar to the concept of *modality streams*, presented in Reference [12]—every node being responsible for a particular property of perceptual data.

For example, the Bluetooth HISS subsystem is designed not to be a simple BT scanner, returning MAC addresses of the devices in range, but it also takes advantage of for example, employee database and conversation history archive. It returns the name and surname of the person, RSSI (Received Signal Strength Indicator) of the BT signal, the direction of movement (coming towards or away from the robot), the history of conversations and possible topics of interest.

The Figure 1 illustrates two groups of external connections made by the core AI engine:

1. Services, depicted inside the green elliptical area on the right-hand side are made for one particular purpose, for example, Speech-to-text service (only). From the engine’s point of view it is important to keep in mind that the services are supposed to be considered as modules, so that it would be possible to replace them with another ones if available, or if their interfacing or their prices change.
2. Smart Sensors, depicted inside with the pink circular area, are the HISS subsystems, customer-related databases and interlocutors-related databases and archives, empowering the subsystems to return substantial high-level information.

During the research and implementation phases, a number of challenges have been encountered, directly affecting the system’s functioning and structure, including the following aspects:

- Communication between components and remote SAS reliability,
- Sensors reliability and redundancy,
- Dynamically changing audio background,
- The volume and scope of information, which can be prepared before the interaction takes place.

As a result of the research a solution for each of the issues have been proposed. The following paragraphs contain a brief discussion of the obtained results.

### 2.1. Communication between Components and Remote SAS Reliability

As presented in Figure 1, all system components communicate with the AI framework engine (main core) which uses a *broker* (an intermediary software module for exchanging information [13]), for a given topic, with/between components, which are subscribed to a specific context. There are many valued message brokers on the market, such as inter alia: Apache Kafka, Nats.io and RabbitMQ. The choice has been made to implement the Apache Kafka broker, while it has a universal protocol, enables to be implemented in any component of system, including sensor nodes. A sensor node consists of a hardware sensor (e.g., a microphone or passive infrared sensor [14]) and a communication enabling Embedded System. A sensor node (ES with a hardware sensor) is designed to take advantage of the Smart Infrastructure, so that it would return high-level result. The reachability of every system component, in particular the HISS subsystems and the cloud-based services, requires additional verification/monitoring, using dedicated passive watchdog modules. One of the most problematic services, which had to be monitored was the Speech-to-text (STT) service, to ensure its availability. The availability validation was performed with sending a simple test stream before the interaction.

### 2.2. Sensors Reliability and Redundancy

Due to the implementation of a fully asynchronous architecture and use of message broker, the sensors were organized into functional groups, depending on the specificity of their tasks.

The groups are being managed with a supervising component, and may contain more than one element, as for example, a group consisting of: infrared sensors, sonars and RFID UHF (Radio-Frequency Identification Ultra High Frequency) gate. This group is managed by a common component (input manager), while they serve the same purpose. The aim is to detect the presence of an interlocutor before the engagement zone of Pepper is being reached (or any other interface agent being used in the particular use case). Such inputs organization, with mixed technologies of data acquisition, may significantly increase malfunction resilience.

### 2.3. Influence of Dynamically Changing Environment

The quality of a Speech-to-text processing is affected by noise generated by devices (called stationary noise) and noise of the external environment (non-stationary noise). Filtering noise without altering the speech quality is a huge challenge. It can be done by using software filters (e.g., WebRTC library [15,16,17]) and/or hardware filters [18]. However, incidental noise may require a sophisticated active noise reduction. The most of the negative impact on the STT quality is caused by non-stationary noise, which is hard to eliminate in environment, but not impossible. The authors confirm that active noise cancellation (against phase emission) enables to lower the noise amplitude to an acceptable level.

### 2.4. Video Input Stream Subsystem (VISS)

**Figure 2:**
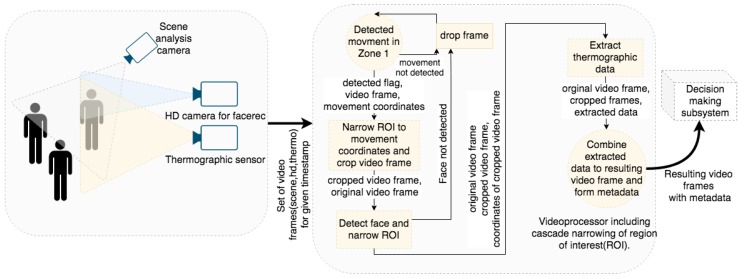
Simplified scheme of the processing of video input streams performed by the VISS.

Despite the fact that the video subsystem is built of up to three cameras, from a macro-scale point of view it is one sensor subsystem which generates input data for the framework. Processing of video frames is done in a cascading manner so that the most resource-intensive operation is in any case successful and ready. If the video acquisition subsystem is equipped with a thermal imaging camera, the video data from the (1) scene camera, (2) high quality camera and (3) thermovision camera are correlated in terms of spatial shift and it is possible to transform the location of the object from the thermovision camera to the scene camera (see Figure 2).

As indicated in Figure 2, the first processing steps are:

1. Comparison of several successive video frames from the scene camera (to determine if any movement have occurred) to report regions of interest (RoI);
2. The appropriate frame is processed by a face detection algorithm, resulting in RoI coordinates;
3. If a thermovision camera is installed, the warmest pixel within every RoI is taken;
4. If a database of employees is installed/handled, the vision subsystem enables to take advantage of connecting to the enterprise’s database and perform face recognition and additional tasks (such as e.g., working time reporting within the employees database);
5. The returned information include: whole video frame, RoI image of every face, any metadata information on detected face objects—transferred from video subsystem to the framework.

The cascade narrowing of the analyzed image results in ability to maintain high processing framerate (similar to the source stream).

### 2.5. Thermovision Subsystem (2020-03 Coronavirus Update)

The latest up-to-date sensor subsystem being developed for the framework, the thermal analysis subsystem, is a good example of the benefit of using the proposed system architecture (a custom AI engine, a humanoid robot and external sensor nodes).

The COVID-19 pandemic forced the company to design a subsystem, which would enable to acquire additional information about the customers—their body temperature. The idea of optional external subsystems turned out to be a great advantage of the proposed solution, proving that designing and adding new subsystems and handling the additional data is relatively easy.

**Figure 3:**
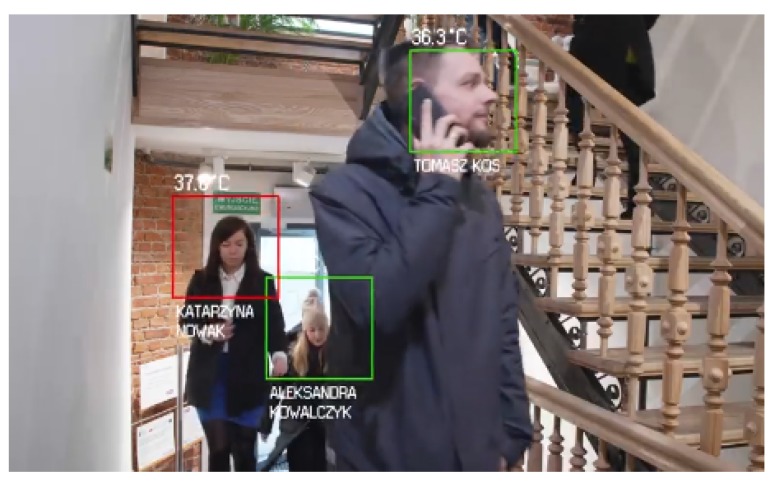
Sample image from the video datalog of the system, visualizing the data acquired by the thermal imaging sensor subsystem.

In Figure 3 a sample image captured from the video datalog system was presented, which illustrates the data obtained with the implementation of the thermal imaging sensor subsystem.

### 2.6. Feedback Loop between Microphones and Speakers

Due to the separation of voice input and output paths, voice connections performed using a PSTN (Public Switched Telephone Network), require adequate synchronization and echo cancellation because of the feedback loop between microphone(s) and speaker(s). However, using a broker, which support streaming assures that the voice output device (in this case: robot Pepper) offers easy access to its input (microphones) stream, which in turn makes it possible to implement echo cancellation. The authors have successfully solved this problem by applying echo cancellation algorithm included in the WebRTC library [15,19].

### 2.7. RFID and Bluetooth Subsystems’ Capabilities After Connecting to Smart Infrastructure

The main advantage of using external HISS subsystems, except modularity, is that the interlocutor can be identified with a set of parameters such as:

1. Object class (animal, luggage, thing, human, child, etc…);
2. Interlocutor class (employee, client, visitor, postman, etc…);
3. Gender, age, and other parameters, before the actual interaction takes place.

One of the robust and convenient means of the person identification is with using an RFID UHF gate (while all the employees have their RFID tags for door access) and Bluetooth (while the conversation history can be maintained for non-employee interlocutors).

**Figure 4:**
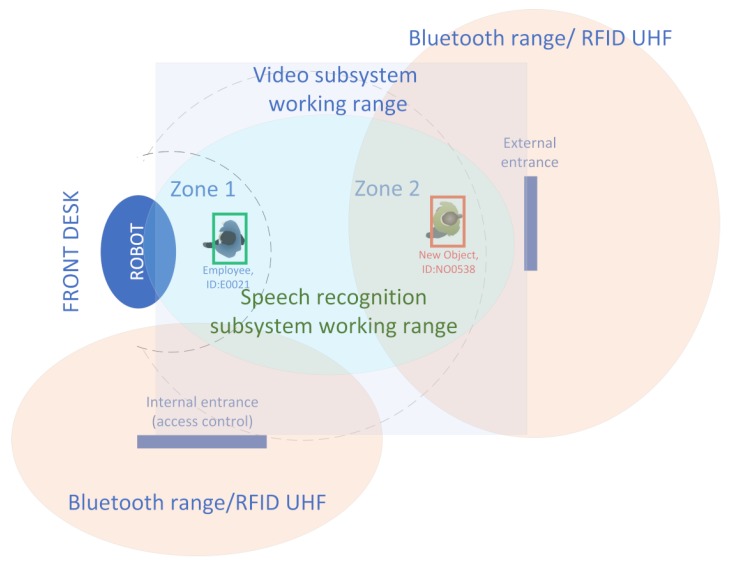
Visualization of the zones for different identification tasks and methods.

Figure 1 presents the basic idea behind the designed system conception, applied to the identification of people arriving at the front desk [20]. The zones in which the individual identification tasks are carried out were marked out. Information can be acquired from many sensors, including cameras and motion sensors. The video module is responsible for visual detection (see Figure 4) of a person in zone 2, for tracking such an object/person, and for identifying its features/parameters, enabling the possibility of exact identification or at least classification in zone 1.

The following subsections include a brief practical description of the proposed concept of the system, as well as some of the remote sensor nodes, which may be used for the system’s capabilities extension.

### 2.8. Smart Sensors and Smart Infrastructure

The proposed system architecture benefits from the smart sensors and smart infrastructure conceptions. The use of external/extrinsic smart sensors improves the quality and efficiency of the individual subsystems, by outsourcing a specific recognition task to a specialized subsystem. The core idea of extending the system’s capabilities by the use of external smart sensors can be explained using the following arguments:

- Data acquisition (e.g., sound recording for speech-to-text subsystem) can be performed using high-end equipment instead of the robot’s built-in microphones, offering better sound quality and better localization of source/interlocutor;
- Integration of the acquisition sensor node within a standalone IoT (Internet of Things) subsystem (thus the word ”extrinsic”) results in modularity—the external acquisition subsystem can be than combined with any kind of robot or interface, moreover, the robot can easily be replaced with another one while it has no custom modifications;
- The IoT sensor subsystem’s integration with the Smart Infrastructure enables to perform some preliminary data processing based on the database records of the company (therefore the word ”smart”), offering much more useful data than the raw output of a particular sensor/device—an RFID gate can provide an information about the ID of a coming employee [21], while a smart RFID subsystem would be able to communicate with the employee’s database and provide the employee’s name, gender, conversation history, and so forth.

The currently developed system includes inter alia the following extrinsic smart sensor subsystems:

- RFID UHF gate (for employee identification);
- Bluetooth scanner subsystem (for the purpose of identification of employee and frequent visitor);
- Speech recognition external subsystem (for sound acquisition, silence detection, and Speech-to-Text processing using Google Cloud Services);
- Face recognition external subsystem (for image acquisition, object/interlocutor detection, face detection, face tracking and face recognition).

The operating methodology of the proposed framework is its ability to (1) acquire numerous inputs concurrently, using independently the proposed autonomous IoT-based Smart Subsystems, and (2) to compose valuable and rich input data for the AI engine, using the data acquired by subsystems as well as additional information from Smart Infrastructure of a particular client/company.

## 3. Results

**Figure 5:**
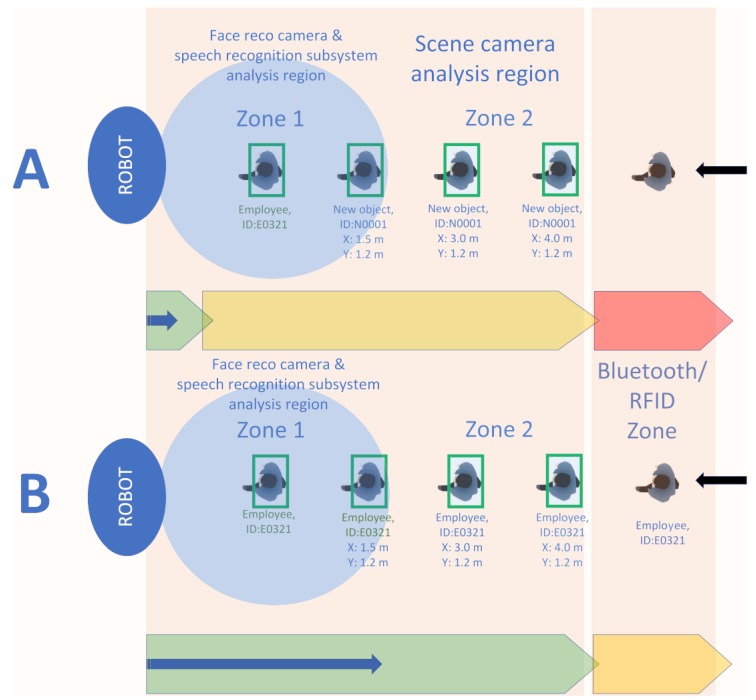
Comparison of mechanisms for obtaining information regarding interlocutor. Use case (**A**)—no RFID or Bluetooth signals available. Use case (**B**)—RFID UHF and Bluetooth subsystems are enabled. Red wide arrow means no information about incoming object/person, yellow wide arrow means partial information about object/person, green wide arrow means detailed information about object/person and blue narrow arrow means the possibility to start personalized interaction between robot (AI framework agent) and interlocutor.

The prototype system has been developed with the implementation of the proposed framework. The testbed for the prototype is the front desk office at the headquarter of the Weegree company (Poland), and it was launched in mid-2018. The main task of the robot is to redirect customers to appropriate departments or employees. The system’s engine detects presence of a person (see Figure 5) and tries to assess/identify the person’s needs. The system is able to make use of existing information systems Smart Infrastructure by obtaining information on the location of departments, checking the availability of selected employees or ongoing events (job interviews, meetings) and the system is able to contact or inform employees by voice or text message.

The Pepper robot is a continuation of robotic system architecture developed by Aldebaran (now SoftBank Robotics) primarily applied in a small humanoid robot NAO [22]. Pepper is significantly larger than NAO, which enhances the interaction experience. Pepper is equipped with a touch tablet, which makes the man-machine communication even easier—the people arriving at the front office seemed to naturally focus their attention on the tablet, although it is neither the first, nor the main, nor the most attractive of robot’s communication channels. Man-machine communication of the Pepper robot can be carried out in many ways, such as inter alia using sound/voice channel, visual channel (touch-sensitive tablet) and nonverbal communication (robot’s posture and gestures).

The prototype of the proposed framework, implemented using the Pepper robot as the hardware base, has proven to be successful, while the customers report to have the impression of actually talking with the robot, despite the fact that the robot is admittedly only a sophisticated interface for the framework to present selected data that come from numerous smart sensors and information systems. The User Experience (UX) of interaction with robot is in part a consequence of its good (apparently sufficient) capability of efficient man-machine interaction—not only by using voice (natural language) [23] and graphical interface, but also by using nonverbal communication, that is, gestures, in various combinations and intensities, depending on the purpose of the message generated by main AI core module.

If the robot is expected to be more versatile, more human-like in its communication, some of its technical parameters may be not sufficient, especially the computational power (Pepper Y20V16 [24] is equipped with ATOM 1.9 GHz quad core, 1 GB RAM, 2 GB Flash, and 8GB Micro SDHC for its Linux-based operating system). Controlling the Pepper robot’s resources can be implemented using a modular approach. To be able to use the Pepper as a front desk officer, the authors have used some of the Pepper’s software modules:

- The ALAnimationPlayer module (responsible for gesticulation, enhancing the robot’s expression);
- The ALTablet module (responsible for obtaining information from the user by the use of touch panel, and for displaying text/graphics), the ALTextToSpeech module (for generating speech),
- The built-in ALSpeechToText module (converting human utterances into text data),
- The ALVideo module (for processing the visual information about the robot’s environment).

The last two, of the mentioned above were not efficient enough for the purpose of this work, therefore the functionality of those modules was replaced/enhanced with the implementation of proprietary modules and services.

In order to provide fluent man-machine communication, one of the key challenges is efficient conversion of natural speech into equivalent text. The built-in Pepper’s speech recognition module is based upon the Niuanse software. It offers only a few languages to choose from, and the embedded version of the library is able to recognize only phrases from a predefined set. Therefore, for recognizing any (not belonging to a predefined set) messages, this solution is insufficient, and so the authors have decided to replace it with libraries/services with greater capabilities in order to meet the requirements of a real natural language conversation. This is particularly important and useful for artificial intelligence applications, which use natural language communication. Such systems are often equipped with additional/external sensors with better technical parameters. A professional microphone, with properly selected characteristics, acquires the interlocutor’s voice much better than the one that is built-in into the robot, an external camera provides a wider angle and/or greater quality/visibility than the stock camera built into the robot and will offer a better input to the system.

**Figure 6:**
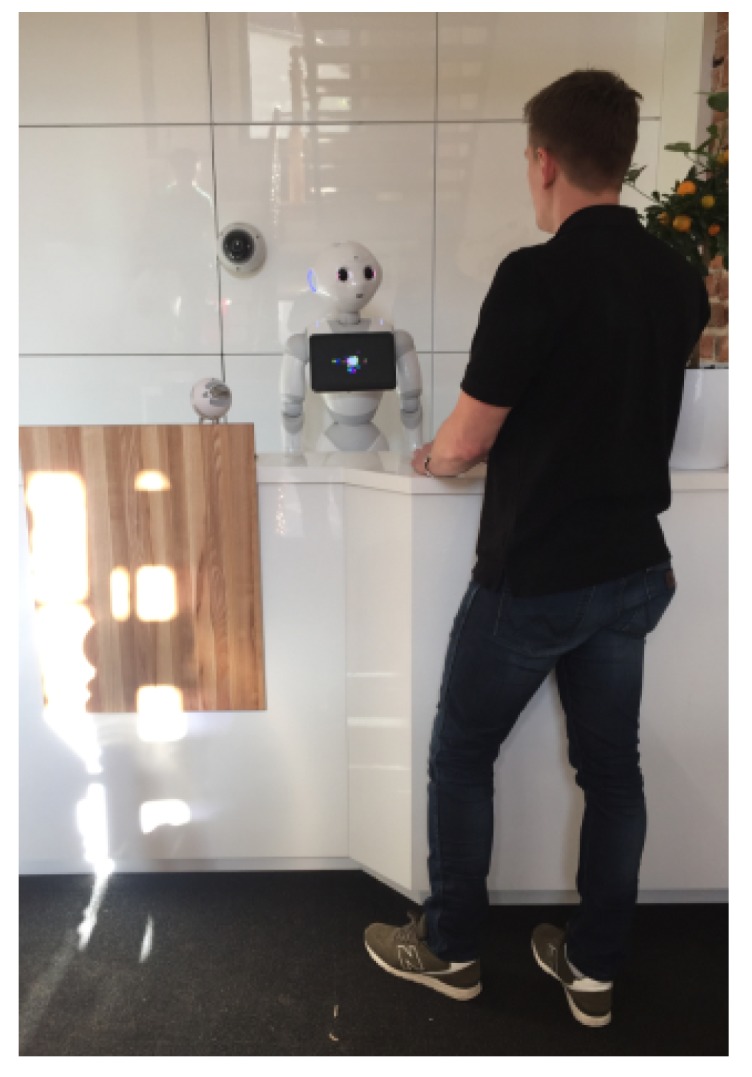
Real-world implementation of the proposed concept. Prototype system with humanoid robot Pepper ready to work. ©2020 IEEE. Reprinted, with permission, from [20].

Having the real-world prototype (see Figure 6) of the system (designed, assembled and programmed) enables many interesting research areas, including:

- Human reactions and attitude to interacting a robotic system,
- Efficiency of the proposed solutions,
- Effectiveness of communication depending on the selected information channel,
- Marketing and image impact of the proposed solutions,
- Feelings and satisfaction of interlocutors/clients,
- The impact of interference on the operation of the system,
- Efficiency of voice data acquisition for Speech-to-text processing in noisy environments,
- Efficacy of video data acquisition and optimization of processing for face detection and recognition,
- Effectiveness of video data acquisition and optimization of processing for object tracking focusing on occlusion and recovery of lost object.

## 4. Discussion

This chapter includes the evaluation of two of the designed subsystems: Bluetooth-based Human Identification Smart Sensor (BT HISS) subsystem and speech recognition external subsystem.

All employees of the Weegree company have RFID tags to access their rooms or building sections or the main entrance. For this reason an RFID gate would be enough to identify an incoming person. However, there are some situations when people do not have RFID tags and these include the following:

- A group of employees is leaving for a lunch and not all of them have RFID tags;
- An employee brings a guest person [25] to the company building;
- A delivery man, a postman, etc…
- A customer.

In the above listed situations it would be also be interesting to know if the person has any conversation history, any rooms or employees to ask about. For this reason the authors have designed BT HISS subsystem, which enables identification of a person by using a modified Bluetooth scanner. The node is built upon Raspberry Pi 4, which has a Cypress CYW43455 Bluetooth 5.0 interface integrated and uses the stock PCB (Printed Circuit Board) antenna. The node is placed near the entrance (see Figure 4 and Figure 5) and it searches for the known Bluetooth addresses. Running a full broadcast-based scan takes way too long (over 3 seconds), for this reason the BT device discovery is not done using a regular scan. The authors assume that the MAC address of the BT device is already known (has been registered in the employees database or discovered using a BT full scan). Knowing a particular MAC address gives the opportunity to detect the device’s presence without the need to pair the Bluetooth devices, moreover, the authors managed to get the signal strength (RSSI) without the need to interrupt the person. Detecting multiple devices of the same person or multiple devices of a group of people is possible and working. The current implementation uses the BlueZ Bluetooth library and a Linux shell script calling ”hcitool cc” (connect) and ”hcitool rssi” (detect signal strength) commands.

**Figure 7:**
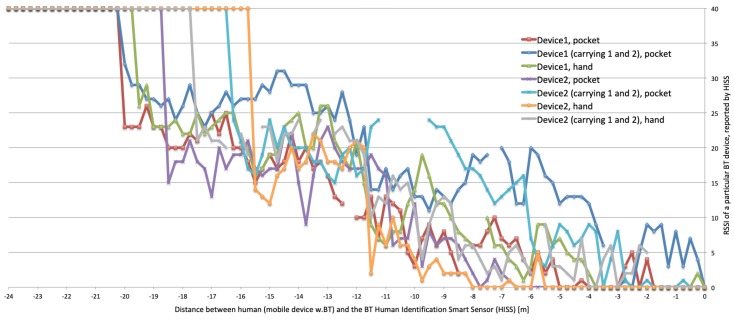
Bluetooth usability test—7 different test scenarios of a BT device carried towards the Bluetooth-based Human Identification Smart Sensor (BT HISS) node. X axis—distance in meters, Y axis—RSSI as a relative index (0 = best quality).

Figure 7 shows exemplary measurements of the RSSI signal strength of mobile devices (smartphones) carried by employees coming from outside the building towards the BT HISS node. The X axis shows the distance to the BT HISS node:

- 25…20.5 meters from gate—outside; then metal door;
- 20.5…11.6 meters from gate—a corridor; then metal/glass door;
- 11.6…0 meters from gate—a corridor and open space.

Please note that the RSSI level indicates the relative quality of the wireless connection, however some manufacturers implement the RSSI as the power level (expressed in dBm, negative values), while other manufacturers understand the RSSI as a relative index (positive values, with 0 as the best quality) in accordance with the IEEE 802.11 standard [26].

The Figure 7 clearly shows these two barriers—metal door at 20.5 and glass/metal door at 11.6 m away from the gate. The initial state of the RSSI is ”device not present” which does not indicate any particular value, but it was visualized in the figure to show when the first contact took place. During the measurements sometimes the signal was lost, which is depicted as missing measurements. What’s interesting, the first device/brand was detected much earlier than the second one, however the second one seems to have more powerful signal while the RSSI reaches level 0 much earlier than the first device.

Although it was possible to calculate approximate distance (in meters) by implementing an approximate regression model based on the measured values, the authors did not need to express the link quality in relation to distance, but to the remaining time before the interaction takes place.

The Figure 7 turned out to be very important for the real world implementation. Although the best RSSI level is about 4 to 8 meters before approaching the BT HISS node, so there is no necessity to wait for the person to come so close. It is enough that the BT node detects presence of the approaching person, so the actual working range is about 16 m. If the BT HISS node gate is located/placed 16 m before the robot, it gives 32 m between first connect and distance = 0. A person walking for 32 m might sound like a lot of time to react, but unfortunately there is a problem/challenge with the current implementation of the connect command, that it takes much longer if the BT device is not present (it has timeout implemented in seconds, not milliseconds). For this reason the node is able to probe a limited number of BT devices.

Therefore, the authors suggest to use RFID UHF HISS node for employee identification and BT HISS node for probing incomers that will not be identified by the RFID subsystem. Practical tests performed at the Weegree company gave a very promising results.

One of the most important intelligent subsystems enabling the interaction of a robotic system with a human is the Speech recognition subsystem, which not only acquires sound (including speech) by the use of built-in or high-end microphones, but also takes care of robust audio packets transmission, STT service switching, watchdogs, and returning the alphanumeric form back to the AI engine.

This section includes the discussion on critical parameters of the proposed audio subsystem and the indication of significant changes, which should be made in order to improve the proposed system’s reliability. Regardless of the physical implementation of the prototype of the proposed system, there are two speech-related critical components of great significance for the final product to be successful. The first one concerns the challenges of Speech-to-text conversion and more precisely its efficiency, whereas the second one concerns the response time to the query sent to the database (chatbot) system. The interaction between a robot (AI engine) and a human is possible immediately after the person is recognized and enters zone 1 (see Figure 4).

This zone can be divided into three subzones depending on the distance of the interlocutor from the robot: far, medium and near the robot. According to Reference [27] people feel comfortable when the man-machine interaction distance is between 0.45 m and 3.6 m, however in the prototype implementation presented in Figure 6 the observed preferred distance was compliant with the Personal Zone, defined in Reference [28], that is, 0.45 m–1.2 m. Figure 7 shows that the RSSI levels are satisfactory below 15 m, and the BT discovery/scan can be performed.

**Figure 8:**
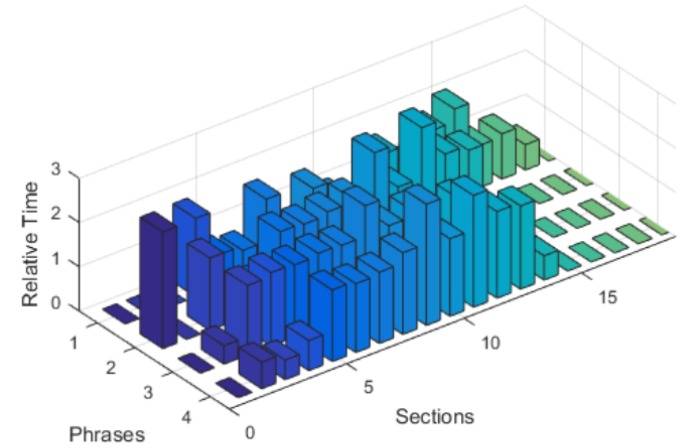
Relation of relative Google Speech-to-text service response times after sending the last frame of the audio speech data to Google.

The Table 1 and the Figure 8 relate to the study of the operation of such a system in real-life conditions for different speaking distances, for a predefined set of various text phrases, using the built-in microphones of the Pepper robot. Phrases 1 to 4 were spoken from the shortest (yet comfortable) distance. Phrases 9 to 12 from a considerable/boundary (border of **zone 1**) (yet comfortable) distance, and phrases from 5 to 8 were spoken from a distance in between of these. All of the transcripts within respective phrase groups: “1 to 4”, “5 to 8” and “9 to 12”, are identical, however, slight variations in the statements uttered by different people/volunteers were observed. While the results of the Google Speech-to-text service varied slightly (transcripts, confidence level, operation time, transmission time), there were also request-response time variations of a basic chatbot engine. For about 200 processing instances of utterances being converted to text, there were 8 values of waiting time (waiting for an answer from STT service) with a value definitely greater than typical values. Including these utterances/measurements significantly increased the average value of the response time of Speech-to-text service and greatly increased the dispersion value of the response time. These values can be found in the last column of Table 1. Values in the preceding columns were calculated, excluding the very high STT response time values, treated here as a measurement error.

where:

- AVSpeechToText—average values of the Speech-to-text service response time, after sending the last frame of the speech audio data to the cloud;
- DispSpeechToText—the value of dispersion for these response times;
- AVChatbot—the average value of the robotic system’s database response time;
- DispChatbot—the value of the dispersion for these response times.

The obtained mean values of STT times (Table 1) show that the processing system can be used in the whole zone 1 without significant loss of system properties.

The chatbot/database subsystem processing time analysis shows a relatively small variation in the response times obtained, although the largest response times are significantly greater than typical ones. After a comparative analysis of relative results obtained for the STT processing and for the database subsystem response times, it can be concluded that in typical cases the maximum waiting time for obtaining an alphanumeric answer (using the Google Cloud Speech-to-text service) does not exceed three times the average value of that time, while for the database system it even reaches ten times the average time. This clearly indicates the need to optimize the operation of database systems in terms of normalizing the response obtained from such systems.

The Figure 8 illustrates the time measurements of the STT service response time. Analysis of the Figure 8 (depicting the presentation of STT processing) shows a clear differentiation of the response times of getting answers for very similar statements of different people made at the same distance. To standardize the obtained results, the results regarding the response time were presented as the multiplicity of the average response time. The relative time shown in the figures is always the ratio of the real time and the corresponding mean value of the time (Table 1).

The analysis of Figure 8 clearly indicates a significant diversity of Google Speech-to-text service response times after sending the last frame of the audio speech data to Google. This applies even to the same phrases spoken in the same conditions but by different people. However, in each case, despite the different response times, the maximum response time does not exceed three times the average time.

### System Prototype Evaluation

Nowadays, many companies are interested in implementing automated systems supporting customer service [2,29,30,31]. The use of a humanoid robot can be a natural extension of methodologies of attracting customers attention. During the testing of the prototype system in the Weegree front desk application it was noted that the robot should initiate contact with people because many of new clients/visitors are surprised by the fact that they can communicate in a very natural way with a robot. It is probably a matter of novelty of the flawless man-machine communication using natural language [32]. However, if the communication (interaction with the robot) is already taking place, people listen to his messages with similar attention and effectiveness as in the case of information from human [33,34].

The proposed system structure combines many services, actuators and sensors. The modular structure of the system enables easy system expansion or replacement of existing modules with new versions.

## 5. Conclusions

During the design and implementation of the robotic front desk officer (using the Pepper humanoid robot as the hardware) in Weegree company, a number of challenges were encountered. One of them concerned the robot’s working space as well as the humanoid robot itself—due to the desk position and limitations of built-in sensors of this particular construction.

The actual study focused on designing a conception, framework, and exemplary implementations of sensor nodes (modules) responsible for:

1. Detection of the presence and for initial identification of a human interlocutor before the actual interaction starts;
2. Shadowing those functionalities of a robot, which do not have sufficient quality/precision, for example, audio acquisition.

The proposed and implemented Human Identification Smart Subsystems (including the proposed Bluetooth HISS subsystem) prove to be successful and useful in a practical implementation. Also the proposed Speech Recognition subsystem is a promising replacement—the processing time required to obtain a SpeechToText transcript is at an acceptable level, and the quality of the transcripts obtained is also satisfactory.

Knowledge acquisition in man-machine interaction is perceived as a process, which takes time. This paper offers a new insight into the process, while it succeeds at acquiring preliminary information about the interlocutor before the interaction starts. Due to the usage of Smart Infrastructure and HISS subsystems the knowledge acquisition becomes a dynamic process and it results in having more time to prepare a conversation strategy by the AI framework engine.

The prototype developed by the authors proves that the proposed conception implemented in a humanoid robot front office use case creates many new possibilities. It also seems particularly promising at the current stage of development of humanoid robot technology in information and advertising applications. The area of applications is expected to grow over time, and humanoid robots equipped with complex systems will become able to enter into application areas currently unavailable to them.

The main aim of this work was to extend already existing humanoid robots with the implementation of various smart systems, which enable verbal, non-verbal and sensor-based interaction. It could also be considered as well as social as service robots while having various smart sensors applied.

The innovative part of this work include: (1) the idea and a prototype implementation of the HISS system architecture (i.e., a custom AI engine, a humanoid robot and external sensor nodes), and (2) exemplary sensor subsystems to be used with the above mentioned smart sensor within the information path, was, fully proposed and developed by the authors of this work.

### Future Work

Currently the authors are implementing the BeamForming algorithms within the Speech recognition smart sensor subsystem to be able to provide multiple text outputs, depending on the speaker/interlocutor, if multiple interlocutors interact with the robot.

The authors are also designing a new sensor subsystem for the framework—the thermal analysis subsystem—which acquires the information about body temperature of people coming to a location/office.

Next research areas and topics include: User Experience (interlocutors’ attitude, reaction and opinion) [1], and detection of abnormality and uncertainty within conversation patterns [35,36].

## Figures and Tables

**Table 1:** Values of average times and dispersion of response time of the Speech-to-text service and the robotic system database (phrases 1–4—minimal comfortable speaking man-machine distance, phrases 5–8—average distance, phrases 9–12 maximal comfortable distance).

	Phrases 1–4 Spoken from the Shortest Distance [s]	Phrases 5–8 Spoken from the Medium Distance [s]	Phrases 9–12 Spoken from the Zone Boundary Distance [s]	All Phrases [s]
AVSpeechToText	0.1688	0.1911	0.1965	0.5861
DispSpeechToText	0.1476	0.1911	0.1449	3.4876
AVChatbot	0.3305	0.2663	0.2813	0.2695
DispChatbot	0.4242	0.2646	0.4124	0.3699

## Abbreviations

The following abbreviations are used in this manuscript:

HISS	Human Identification Smart Subsystem
RSSI	Received Signal Strength Indicator
SAS	Software As a Service
STT	Speech To Text
ES	Embedded System
RFID	Radio-Frequency Identification
UHF	Ultra High Frequency
BT	Bluetooth
